# Intravenous anti-*P. aeruginosa* IgY-antibodies do not decrease pulmonary bacterial concentrations in a porcine model of ventilator-associated pneumonia

**DOI:** 10.1177/17534259221114217

**Published:** 2022-11-13

**Authors:** A. Otterbeck, P. Skorup, K. Hanslin, A. Larsson, J. Stålberg, H. Hjelmqvist, M. Lipcsey

**Affiliations:** 1 Anesthesiology and Intensive Care, 174469 Department of Surgical Sciences, Uppsala University, Uppsala, Sweden; 2 Section of Infectious Diseases, 214437 Department of Medical Sciences, Uppsala University, Uppsala, Sweden; 3 Section of Clinical Chemistry, 214437 Department of Medical Sciences, Uppsala University, Uppsala, Sweden; 4 Anesthesiology and Intensive Care, School of Medical Sciences, 98837 Örebro University, Örebro, Sweden; 5 Hedenstierna laboratory, Department of Surgical Sciences, 8097 Uppsala University, Uppsala, Sweden

**Keywords:** Anti-microbial resistance, chicken antibodies, hospital acquired pneumonia (HAP), pneumonia

## Abstract

Ventilator associated pneumonia (VAP) caused by *P. aeruginosa* is a cause of morbidity and mortality in critically ill patients. The spread of pathogens with anti-microbial resistance mandates the investigation of novel therapies. Specific polyclonal anti-*P. aeruginosa* IgY-antibodies (*Pa-*IgY) might be effective for VAP caused by *P. aeruginosa.* The objective of this study was to investigate if intravenous *Pa-*IgY decreases the lower airway concentration of *P. aeruginosa* in VAP. We used a double blind randomized placebo controlled porcine model of VAP caused by *P. aeruginosa*. Eighteen pigs were randomized to either receive intravenous *Pa-*IgY or placebo. Repeated registration of physiological parameters and sampling was performed for 27 h. Concentration of *P. aeruginosa* in BAL-cultures was similar in both groups with 10^4.97^ ± 10^2.09^ CFU/mL in the intervention group vs 10^4.37^ ± 10^2.62^ CFU/mL in the control group at the end of the experiment. The intervention group had higher heart rate, cardiac index, oxygen delivery and arterial oxygen tension/fraction of inspired oxygen-ratio, but lower plasma lactate and blood hemoglobin levels than the control group. In summary, in an anesthetized and mechanically ventilated porcine model of VAP, *Pa-*IgY at the dose used did not decrease concentrations of *P. aeruginosa* in the lower airways.

## Background

Ventilator-associated pneumonia (VAP) is a common cause of morbidity and mortality in the intensive care unit (ICU).^1^ *Pseudomonas aeruginosa* is responsible for approximately 20% of VAP-cases and is known to host resistance to several broad spectrum antibiotics.^2^ When caused by multi drug resistant pathogens, mortality in VAP increases dramatically making novel treatment strategies against VAP caused by *P. aeruginosa* an important area of research.^3^

Immunoglobulin Y (IgY) has been shown to have antibacterial properties,^4^ it is a monomeric antibody produced in reptiles and birds. Since these antibodies are present in hen eggs, a common part of the human diet, they are very unlikely to cause adverse immunologic reactions in humans.^5^ Hens inoculated with *P. aeruginosa* produce eggs with yolks containing high titers of specific polyclonal anti-P. aeruginosa IgY-antibodies (*Pa*-IgY), thus providing an inexpensive means of production.^6^ *Pa*-IgY work primarily by binding to the flagella of *P. aeruginosa* promoting phagocytosis by polymorphonuclear neutrophils.^7^ The flagella of *P. aeruginosa* is known to bind to toll like receptor 5 (TLR-5), in turn TLR-5 activates inflammatory pathways by production of tumor necrosis factor-α (TNF-α), interleukin-6 (IL-6), interleukin-10 (IL-10) and interleukin-8 (IL-8).^8,9^ Both experimental and clinical studies have shown that *Pa*-IgY counteract *P. aeruginosa* growth in vivo.^4,10,11^ All these studies deliver *Pa*-IgY into the airways or gastrointestinal tract, intravenous (*iv*) injections of IgY have been studied in mice and dogs. To our knowledge there are no studies on iv *Pa*-IgY in any animal.^12,30^ The assumed safety of IgY, the lack of studies on *iv Pa-*IgY and the need for treatment alternatives in VAP caused by *P. aeruginosa* warrants a study like this one.

Our hypothesis was that *iv* treatment with *Pa-*IgY could decrease lower airway concentration of *P. aeruginosa* in an experimental model of VAP. We used a double blinded randomized large animal model in piglets. Pigs have similarities in physiology, anatomy and immune system to humans and hence constitutes a rational choice of animal for this investigation.^13,14^ This model also allows for invasive monitoring and repeated blood sampling.

Our primary endpoint was group difference in concentration of *P. aeruginosa* in broncho-alveolar lavage (BAL) over time in pigs treated with *Pa-*IgY vs placebo. Secondary endpoints were group differences in respiratory parameters, circulatory function, inflammatory markers, anti-*P. aeruginosa* activity in plasma, kidney function and *P. aeruginosa* concentration in blood and pulmonary biopsies.

## Materials and methods

### Ethical approval

Animals were handled according to guidelines from the Animal Ethics Board (Uppsala, Sweden) and the European Union’s directives for animal research. Ethical approval was granted by the Uppsala animal ethics committee (application C155/14 and 5.8.18-08592/2019). When applicable MQTiPSS-recommendations have been adhered to.^15^ Data is presented according to ARRIVE-guidelines.^16^

### Study protocol

This study is a randomized placebo controlled double blinded experimental animal study. Cross bred Norwegian landrace pigs, 6–8 weeks old, were used. Pigs were randomized using sealed envelopes containing either *Pa*-IgY or placebo (NaCl) in masked test tubes and administered through masked syringes. Group allocation was concealed until after the statistical analysis. The experiments were carried out in an animal research facility with experienced staff and in an ICU-like setting. Piglets arrived from the breeder in the morning and experiments were started after preparation and stabilization of the piglets after approximately 2 h. The experiment was carried out for 27 h. Pigs were excluded before randomization if there were clear clinical signs of infection, respiratory or hemodynamic dysfunction.

Anesthesia was induced with tiletamine/zolazepam 6 mg/kg (Zoletil, Virbac, Stockholm, Sweden) and xylazine 2.2 mg/kg (Rompun, Bayer, Copenhagen, Denmark) followed by 100 mg ketamine (Ketaminol, Intervet, Stockholm, Sweden) and 20 mg morphine (Morfin Meda, Meda, Solna, Sweden) when *iv* access was established. Anesthesia was maintained with 1g pentobarbital (Pentobarbitalnatrium, Apoteket, Stockholm, Sweden) and 32.5 mg morphine mixed in 1000 mL of 25 mg/mL glucose given at 8 mL/kg/h. Muscle relaxation to prevent shivering was achieved using rocuronium 10 mg/mL (Esmeron, MSD, Stockholm, Sweden) infused at 2.5 mg/kg/h. Ringer’s-Acetate (Ringer-acetat, Fresenius Kabi, Uppsala, Sweden) was initially given as a bolus of 20 mL/kg and thereafter as maintenance at 2 mL/kg/h intravenously. Additional morphine and ketamine was administered as needed to keep the animals anesthetized. Clinical signs and a withdrawal reflex to painful stimulus (maintained with the level of muscle relaxation used) to the soles of the feet was used to monitor anesthetic depth. All pigs were mechanically ventilated through a tracheostomy with the following settings: Ratio of inspiratory:expiratory time 1:2, fraction inspired oxygen (FiO_2_) 0.3, tidal volume 10 mL/kg, respiratory rate 25, positive end-expiratory pressure (PEEP) 5 cmH_2_O. The pigs received a central venous catheter, a pulmonary artery catheter, an arterial catheter and a suprapubic urinary catheter. All pigs were administered 750 mg of cefuroxime peri-operatively, to which *P. aeruginosa* is naturally resistant. After preparation and 30 min of stabilization the experiment started. The experiment was carried out with animals lying in the lateral position, changing side followed by alveolar recruitment every 6 h.

Noradrenaline 20 µg/mL (Noradrenalin, Hospira Nordic, Stockholm, Sweden) was started as a continuous infusion at 5 mL/h if needed and increased accordingly to maintain a mean arterial pressure (MAP) > 60 mmHg. At cardiac output < 2 L/min a clinical decision was made to either increase the rate of noradrenaline or to give a 15 mL/kg bolus of Ringer’s Acetate. Normoventilation (PaCO_2_ 4.5–6.5 kPa) was achieved by adjusting tidal volume. Adequate oxygenation (93% < SaO_2_ < 100%) was achieved by adjusting FiO_2_ and for repeated hypoxemia PEEP was incrementally increased followed by a recruitment maneuver. The animals were heated as necessary using heating pads, fluid warmers and covers to maintain a body temperature between 35 and 42°C. Blood glucose < 4.0 mmol/L was treated with a bolus of 20 mL 30% glucose. At the end of the experiment the pigs were sacrificed by injection of 20 mL KCl.

### IgY-production

The method used for production of *Pa*-IgY has been previously described in detail.^4,17,18^ Briefly, hens were inoculated with *P. aeruginosa* and *Pa-*IgY was then purified from the egg yolks. The activity of purified *Pa*-IgY was measured with enzyme-linked immunosorbent assay (ELISA) to yield equally active batches resulting in concentrations of *Pa-*IgY at approximately 10 mg/mL as measured spectrophotometrically. *Pa*-IgY was donated by Immunsystem I. M. S. AB (Uppsala, Sweden).

### Intervention

The pigs were randomized to receive either *P. aeruginosa* + *Pa-*IgY (intervention, n = 9) or *P. aeruginosa* + NaCl (control, n = 9). After recording baseline data and collecting baseline samples a catheter was placed in the main bronchus of the lower right lobe under bronchoscopic guidance. Twenty mL of 10^9^ CFU/mL *P. aerug*inosa (PA-103, ATC 29260, CCUG31589) in log-phase was administered through the right lower lobe catheter. The strain is a virulent clinical isolate from sputum with a type 3 secretion system and exotoxin A production. The strain is tested for when logarithmic growth phase occurs *in vitro* (105 min) and is resistant to pig serum. Immediately after bacterial administration an *iv* injection of either 10 mL *Pa-*IgY 10 mg/mL or 10 mL NaCl 9 mg/mL according to randomization. This dose was then repeated after 12 h due to the decreasing effect of *Pa-*IgY on concentrations of *P. aeruginosa* seen in our previous study.^4^

### Data collection

**Figure 1.**
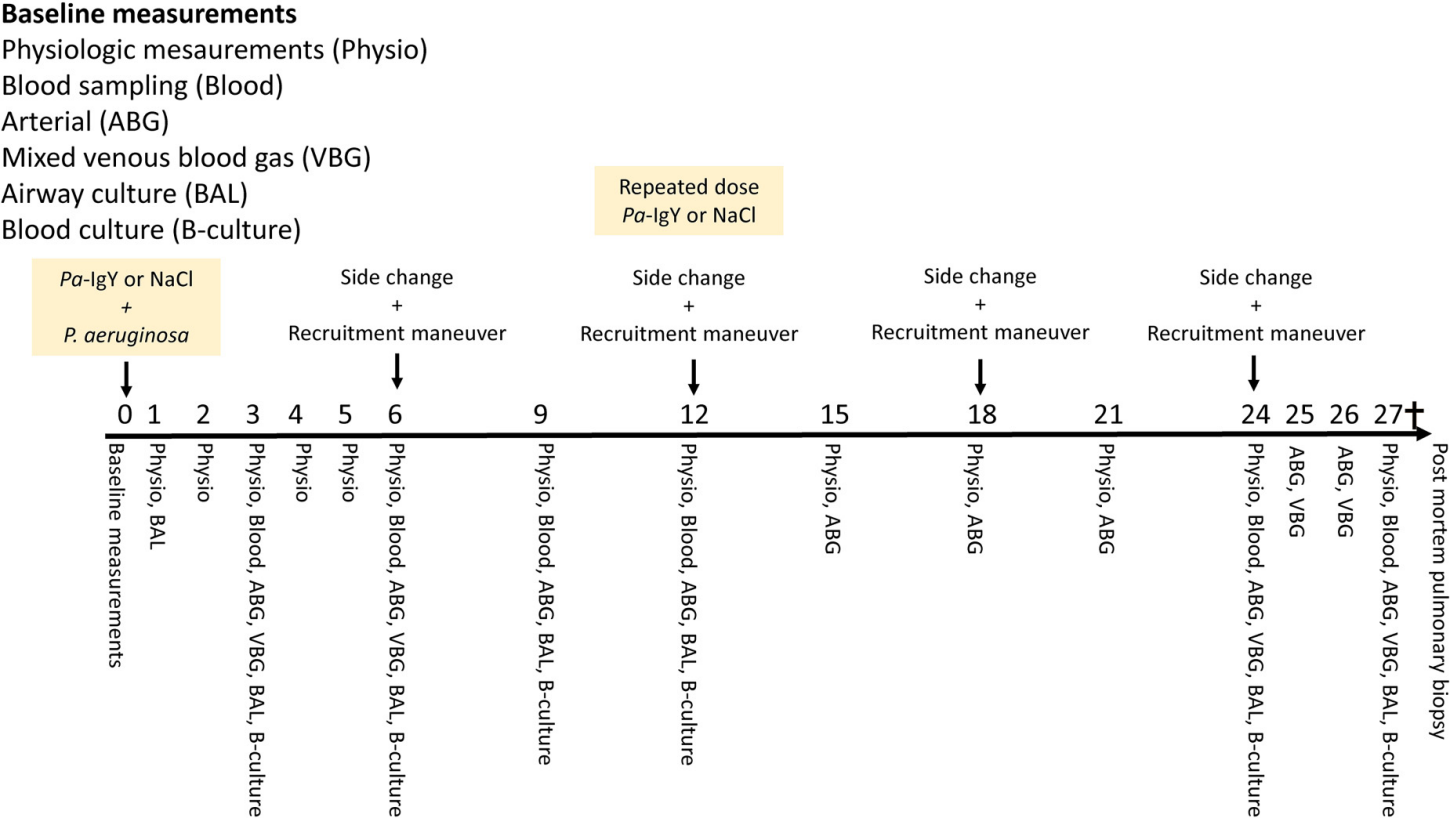
An overview of the data collection during the experiment. Time points for recruitment and rotation to the contralateral side of the pigs is also shown. Administration of study drug and *P. aeruginosa* was done after collection baseline data.

We measured physiological parameters, collected blood samples in ethylenediamin tetra-acetic acid (EDTA)- and litium-heparin tubes and collected urine at predefined time points (Figure 1). These samples were analyzed for complete blood counts, plasma and urine creatinine levels and plasma cytokines including IL-6 (detection limit >125 pg/mL), TNF-α (detection limit >30 pg/mL) IL-10 (detection limit >23 pg/mL) and IL-8 (detection limit >62 pg/mL). Cytokines were analyzed using commercial enzyme-linked immunosorbent assays (ELISAs) for porcine IL-6, TNF-α, IL-10 and IL-8 (DY686, DY690B, DY693B and DY535, R&D Systems, Minneapolis, MN, USA). Plasma from the same time points was analyzed for anti-*P. aeruginosa* activity using ELISA which is a measure of the antibody activity against *P. aeruginosa* (cut-off for background activity <20 mFKU).^19^ Blood gases were analyzed in blood gas analyzers at the animal research facility (ABL835- FLEX Radiometer and OSM3 Oximeter Radiometer). As a surrogate marker for adequacy of microcirculatory flow we used the ratio of the venoarterial difference in partial pressure of carbon dioxide to the arteriovenous difference of oxygen content (ΔpCO_2_/ΔcO_2_).^20^ Airway bacterial cultures were acquired through a BAL at predefined time points consisting of a 10 mL NaCl 9 mg/mL injection with subsequent aspiration in the right lower lobe catheter. One-hundred µL of the lavage was then serially diluted and cultured on cysteine-lactose-electrolyte-deficient agar (CLED) plates to determine the *P. aeruginosa* concentration. Each dilution was cultured on a CLED plate. Blood was drawn from the arterial catheter and 100 µL was cultured on CLED plates in duplicate. Two 3 mm punch biopsies were taken post-mortem from each quadrant of the right lower lobe and then cultured on CLED plates in duplicate after sterile crushing and dilution in 3 mL NaCl 9 mg/mL. All cultures were incubated over night at 37°C and the bacterial concentration was determined with viable count technique.

### Statistical analysis

Data is presented according to normality (assessed in a histogram) as mean ± SD or median (IQR) unless otherwise stated. Differences in baseline characteristics were tested for using an independent Student’s t-test or Mann-Whitney U-test according to normality. All variables except for white blood cell count (WBC), PaO_2_/FiO_2_-ratio and ΔpCO_2_/ΔcO_2_ were log normally distributed and were thus log transformed. The anti-*P. aeruginosa* activity in plasma was log-normally distributed for values above cut-off and was analyzed accordingly. Due to baseline differences in hemoglobin (Hb) levels and apparent differences in ΔpCO2/ΔcO_2_ and TNF-α (Table 1) we indexed all measured variables to the baseline value (eg *n*h Hb/0h Hb) to decrease the risk of type 1 error. Growth of *P. aeruginosa* in BAL was not indexed due to there being no growth in almost all cultures at 0h. To test for differences between groups and over time, general linear models for repeated measures (ANOVA III) was used. Difference over time was tested from 0h and group differences were tested from 1h. A p-value < 0.05 was considered statistically significant, data were analyzed using SPSS (IBM SPSS Statistics for Windows, Version 25.0. IBM Corp) and Statistica (ver 13.2, Dell Inc, Tulsa, USA).

**Table 1.** Baseline characteristics.

	Intervention	Control	*p*-value
Weight (kg)	28.5 ± 0.8	28.4 ± 1.7	0.845
HR (min^−1^)	91 ± 16.7	102 (89–115)	0.08
MAP (mmHg)	89 ± 9	88 ± 9	0.765
Temperature (°C)	39 ± 1.0	39 ± 0.8	0.753
aLactate (mmol × L^−1^)	1.6 ± 0.4	1.4 ± 0.2	0.225
Blood Hemoglobin (g × L^−1^)	85 ± 5	91 ± 3	0.010
Blood WBC (10^9×^L^−1^)	15.6 ± 5.2	16.7 ± 4.1	0.609
Blood Neutrophil count (10^9^ × L^−1^)	7.3 ± 2.7	7.8 ± 2.7	0.657
CI (L × min^−1^ × m^−2^)	3.58 ± 0.87	4.20 ± 0.60	0.097
Static compliance (mL × cmH_2_O^−1^)	33.2 ± 5.6	33.2 ± 9.6	0.992
PaO_2_/FiO_2_-ratio (mmHg)	329 ± 76	359 ± 64	0.376
Plasma Creatinine (µmol/L)	69 ± 12	60 ± 5	0.067
Plasma TNF-α (pg/mL)	400 ± 329	189 ± 141	0.109
Plasma IL-8 (pg/mL)	8.8 ± 7.7	12.7 ± 6.1	0.279

Values presented are mean ± SD or median (IQR). HR = Heart rate. MAP = Mean arterial pressure. aLactate = Arterial lactate. WBC = White blood cell count. CI = Cardiac index. TNF-α = Tumor necrosis factor α. IL-8 = Interleukin 8.

## Results

One pig was excluded before randomization due to a clinically evident soft tissue infection on arrival and one pig was excluded before randomization due to respiratory failure after intubation, both pigs were replaced. Nine pigs were randomized to each group. Two pigs in the control group died of vasoplegic circulatory collapse, one at 24.5 h and one at 9.5 h. One pig in the intervention group died of the same cause at 11 h. The data from these pigs was included as planned until their death.

### Baseline characteristics

The intervention group was similar to the control group at baseline except for levels of Hb (Table 1). The intervention group had lower Hb compared to the control group at baseline, 85 ± 5 vs. 91 ± 3 (*p* = 0.01).

### Bacterial cultures

**Figure 2.**
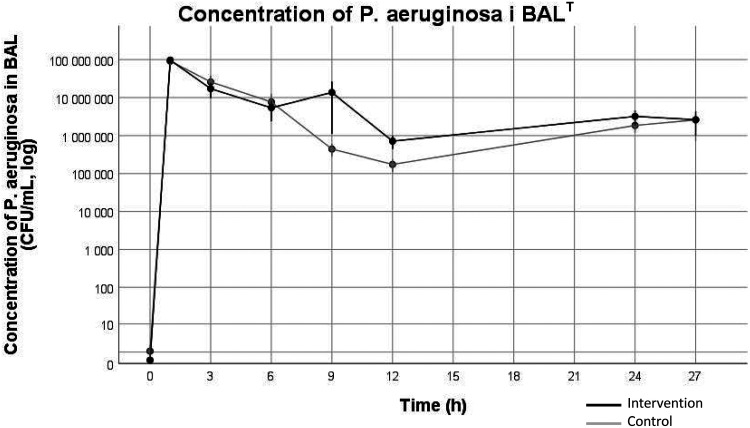
Growth of *P. aeruginosa* in BAL-cultures. Values are mean ± standard error of the mean (SEM). Values are not log-converted, note that the y-axis is a log scale. Black line represent the intervention group. Gray line represents the control group. BAL = Broncho alveolar lavage. Data was analyzed using mixed linear models. T = Significant difference over time (*p* < 0.001).

Bacterial concentration of *P. aeruginosa* in BAL increased over time (*p* < 0.001) in both groups with no difference between groups, peaking at 10^7.89^ ± 10^0.36^ CFU/mL in the intervention group vs 10^7.77^ ± 10^0.52^ CFU/mL in the control group at 1 h. Thereafter BAL *P. aeruginosa* concentration decreased until the end of the experiment (Figure 2). One pig in the intervention group and two pigs in the control group had growth of *P. aeruginosa* before the start of the experiment with BAL concentrations of 20 CFU/mL, 90 CFU/mL and 10 CFU/mL respectively. Growth of *Bordetella bronchiseptica* was seen at the start of the experiment for 5 pigs in the control group, this was not observed in subsequent samples.

All lungs were macroscopically pathological with atelectasis, hemorrhage and purulent secretions isolated to the right lower lobe. There was no difference in concentration of *P. aeruginosa* in pulmonary biopsies between groups, 10^5.09^ ± 10^1.57^ CFU/mL in the intervention group vs 10^5.00^ ± 10^1.72^ CFU/mL in the control group. There was no growth of *P. aeruginosa* in blood cultures.

### Physiological parameters and laboratory analyzes

Heart rate (HR) increased over time (*p* < 0.001) in both groups with higher values in the intervention group compared to the control group (*p* < 0.01). Mean arterial pressure (MAP) decreased over time (*p* < 0.001) with no difference between groups. There was no difference in total noradrenaline dose between groups, 12,386 (118- 24,654) µg in the intervention group vs 2725 (0-9646) µg in the control group. CI changed over time in both groups (*p* < 0.001) with higher values in the intervention group compared to the control group (*p* < 0.001). Systemic vascular resistance index (SVRI) changed over time in both groups (*p* < 0.001) with lower values in the intervention group compared to the control group (*p* < 0.001). DO_2_ changed over time (*p* < 0.001) with higher values in the intervention group (*p* < 0.01). There was no difference over time in lactate and lactate was lower in the intervention group compared to the control group (*p* < 0.01). Hb changed over time (*p* < 0.01) with lower values in the intervention group (*p* < 0.001). ΔpCO_2_/ΔcO_2_ decreased over time (*p* < 0.01) with higher values in the intervention group (*p* < 0.001). Mean pulmonary arterial pressure (MPAP) increased over time (*p* < 0.001) with no difference between groups.

Static compliance decreased over time (*p* < 0.001) in both groups with no difference between groups. PaO_2_/FiO_2_-ratio decreased over time in both groups (*p* < 0.001) with higher values in the intervention group (*p* < 0.01).

Plasma creatinine changed over time in both groups (*p* < 0.01) with no difference between groups.

**Figure 3.**
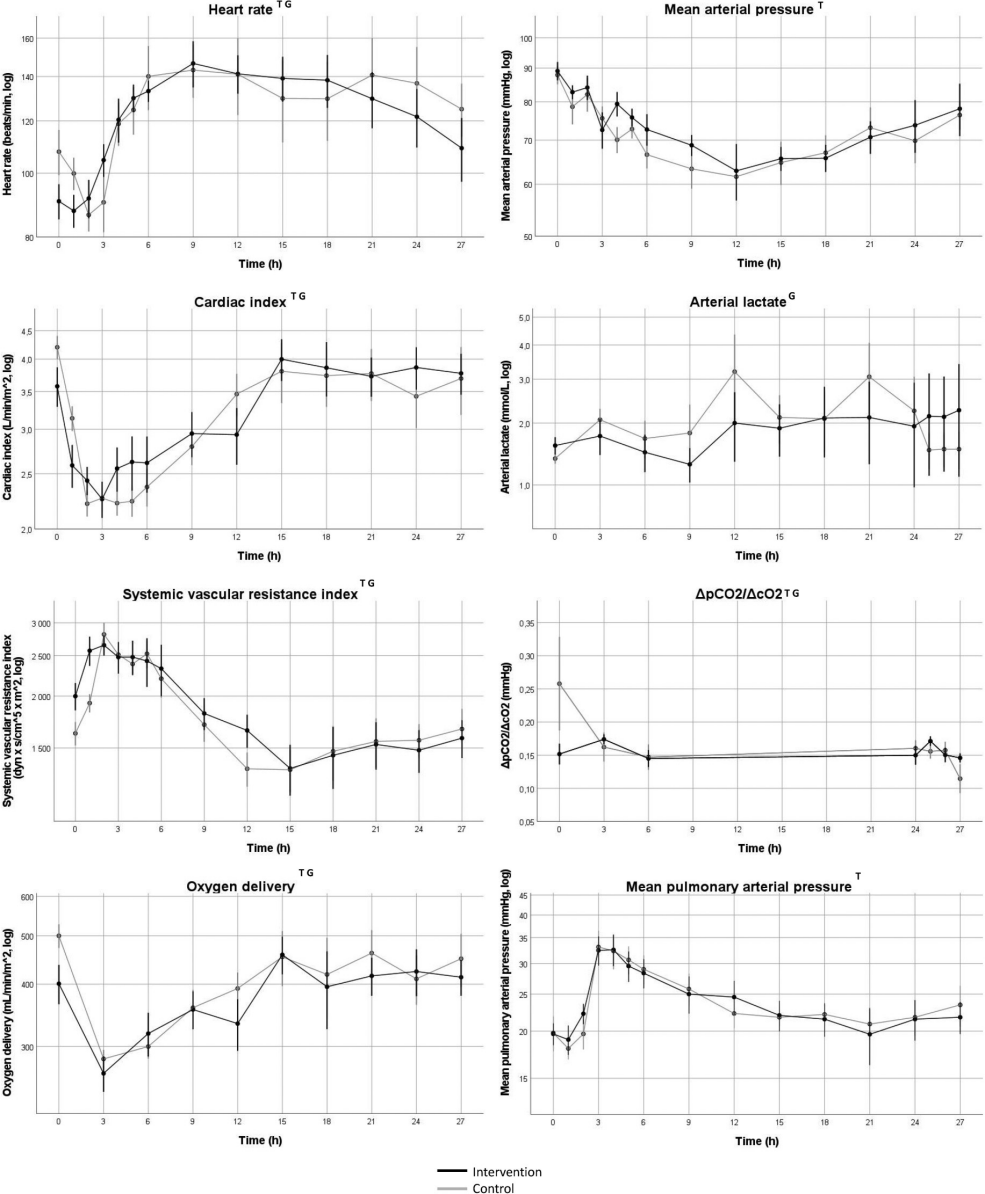
Change in circulatory function over time. Values indicate mean values, error bars denote SEM. Black line represents intervention group, gray line represents control group. log = values were log-converted before analysis and the y-axis is logarithmic. ΔpCO_2_/ΔcO_2_ = venoarterial difference in partial pressure of carbon dioxide / arteriovenous difference in oxygen content. T = Change in variable 0–27 h, *p* < 0.05. G = Group difference after baseline to 27 h, *p* < 0.05. Data was indexed to the 0h-value before statistical analysis with mixed linear models.

**Figure 4.**
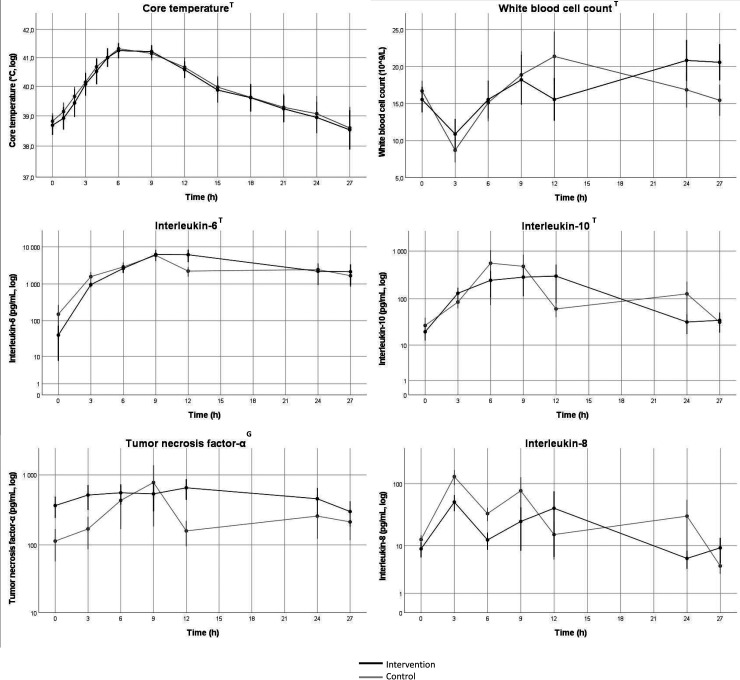
Change in markers of inflammation over time. Values indicate mean values, error bars denote SEM. log = values were log-converted before analysis and the y-axis is logarithmic. Black line represents intervention group, gray line represents control group. T = Change in variable 0–27 h, *p* < 0.05. G = Group difference after baseline to 27 h, *p* < 0.05. Data was indexed to the 0h-value before statistical analysis with mixed linear models.

**Figure 5.**
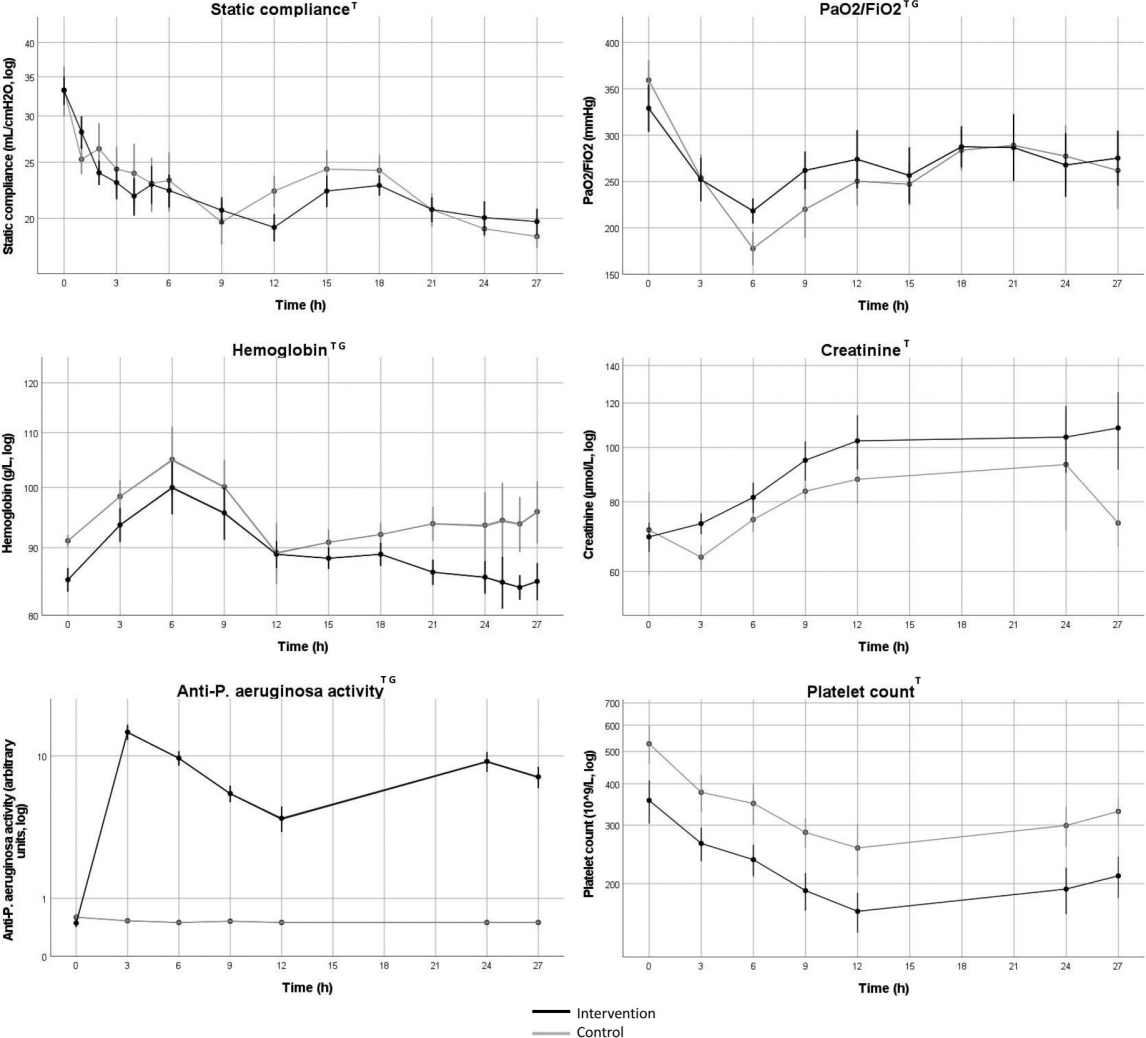
Change in respiratory function, hemoglobin, kidney function, anti-*P. aeruginosa* activity and platelet count over time. Values indicate mean values, error bars denote SEM. All variables except PaO_2_/FiO_2_ were log-converted for analysis. log = values were log-converted before analysis and the y-axis is logarithmic. Black line represents intervention group, gray line represents control group. PaO2 = Arterial partial pressure of oxygen. FiO2 = Fraction of inspired oxygen. Log = values are log converted. T = Change in variable 0–27 h, *p* < 0.05. G = Group difference after baseline to 27 h, *p* < 0.05. Data was indexed to the 0h-value before statistical analysis with mixed linear models.

Core temperature increased and then decreased over time in both groups (*p* < 0.001) with a nadir at 6 h and no difference between groups. White blood cell count (WBC) changed over time (*p* < 0.05) with no difference between groups. IL-6 and IL-10 increased over time (*p* < 0.001 and *p* < 0.05) with no difference between groups. There was no change over time in TNF-α, but values were lower in the intervention group compared to the control group (*p* < 0.05). Values for IL-8 were generally low with a majority of values below cut-off. However, there was a marked increase in IL-8 after three hours in the three pigs that died during the experiment. Platelet count decreased over time (*p* < 0001) with no difference between groups. IL-8 increased in the control group over time but not in the intervention group (*p* < 0.05), analyzed using Friedman’s ANOVA (Figures 3, 4 and 5).

### Anti *P. aeruginosa* activity

There was an increase from baseline (*p* < 0.01) in anti *P. aeruginosa* activity with higher values in the intervention group compared to the control group (*p* < 0.001).

## Discussion

We have used a porcine model of VAP caused by *P. aeruginosa* to test the hypothesis that treatment with *iv Pa-*IgY reduces the concentration of *P. aeruginosa* in BAL-cultures. There was no difference in concentrations of *P. aeruginosa* between groups. The intervention group had higher HR, CI, DO_2_, ΔpCO_2_/ΔcO_2_, PaO_2_/FiO_2_ but lower SVRI, plasma lactate and blood hemoglobin levels than the control group. Intravenous *Pa-*IgY has not been studied before and there were no signs of severe toxicity in our experiments.

The macroscopic changes seen in the lungs post-mortem showed signs of pneumonia. Inflammatory markers increased and respiratory parameters deteriorated until 6 h, this gradual change is postulated to represent the development of pneumonia. Animals were hemodynamically unstable and were therefore given high doses of noradrenalin. Three pigs died from circulatory collapse. BAL-concentrations of *P. aeruginosa* increased immediately after instillation and remained high without group differences. There was growth of *P. aeruginosa* in three pigs prior to the start of the experiment. This growth might have been due to laboratory contamination, contamination during surgical preparation or actual airway growth. The pigs respiratory- and inflammatory parameters of these pigs were not different from the other animals at baseline. The magnitude of growth was so small that it is very unlikely to have affected our results.

These results are in line with previous work in our lab where a bronchial injection of *Pa-*IgY did not reduce pulmonary concentrations of *Pa-*IgY.^21^ However, they contradict our first study where a nebulization of *Pa-*IgY decreased the concentration of *P. aeruginosa* in the large airways.^4^Our results contradict the results from trials on patients with cystic fibrosis, mice with pneumonia and in vitro experiments.^7,10,11^ In the circumstances of those experiments, where *Pa*-IgY was not administered intravenously, *Pa*-IgY appears to have an effect on the growth of *P. aeruginosa.* Our lack of effect might be explained by the dose of *Pa-*IgY and the delivery of *Pa-*IgY. In this study the dose of *P. aeruginosa* was high compared to other models of VAP caused by *P. aeruginosa.*^22^ We chose this dose because it has previously produced reliable pneumonia with hemodynamic derangement and reliable growth in BAL-cultures (our pilot experiments). The bacterial concentration in BAL from patients with VAP is generally 10^4^ CFU/mL^23^ which is 0-3 orders of magnitude less than in our study. It cannot be ruled out that *Pa*-IgY might have an effect either when the bacterial load is lessened or if the dose of *Pa*-IgY is increased. Compared to our previous study of *Pa-*IgY in tracheal colonization,^4^ where there was an effect on growth of *P. aeruginosa*, our dose of *Pa-*IgY is 4 times higher while BAL concentration of *P. aeruginosa* is 3–4 orders of magnitude higher. Intravenous drug delivery is a well-established and commonly used method of drug delivery in intensive care which is why we chose to use *iv Pa-*IgY in this study. Although the *iv* route guarantees delivery of the full dose to the blood stream the delivery of *Pa-*IgY to the infection site is not guaranteed. When comparing the dose of Pa-IgY to the dose of *P. aeruginosa* we used a ratio of 2.5 *×* 10^−9^ per CFU of *P. aeruginosa*. This is higher than in the study by Thomsen *et al*. of murine pneumonia where they used 7 *×* 10^−10^ mg/CFU. Immunoglobulins are large molecules and this likely affects the delivery to the lungs. In our previous study with intrabronchial injection of *Pa-*IgY we detected anti-*P.aeruginosa* activity in plasma which was a magnitude lower than in this study.^21^ This might imply that only a small fraction of *Pa-*IgY crosses the capillary-alveolar membrane. Immunoglobulin G has similar characteristics in that alveolar levels are generally 1000 times lower than in the circulation.^24^ This is not surprising since immunoglobulins generally require active transport by binding to Fc-receptors to be transported across membranes.^25^ *Pa-*IgY does not bind to the mammalian Fc-receptor, low alveolar levels are therefore likely.^6^ A contributing factor might have been heterogeneous blood flow in the lung. Although the lungs receive the entirety of cardiac output, distribution of that blood flow is heterogeneous. Shunting of blood flow away from the infection site due to hypoxic and inflammatory vasoconstriction likely decreases delivery of *Pa-*IgY,^26,27^ this effect should however become less important over time. Delivery of *Pa-*IgY to atelectatic regions of the lung should be less of an issue with *iv* delivery than administration through the airways. An estimation of anti *P. aeruginosa* activity in BAL would be of great interest but the ELISA method we used in blood is not reliable when large concentrations of *P. aeruginosa* are present.

Flagella of *P. aeruginosa* are known to bind to TLR-5 as part of the innate immune response leading to the production of several cytokines.^8,9^ We saw a group difference in TNF-α levels over time which is in line with the possibility of *Pa*-IgY binding to flagellin and exerting an anti-inflammatory effect.^28^ However, this is contradicted by the fact that we saw no group difference in other cytokines even though we did have an increase over time in IL-6 and IL-10. The difference in IL-8 is difficult to interpret due to most values being below cut-off. We saw increased anti-*P. aeruginosa* activity in plasma in the intervention group compared to the control group, this is expected with *iv* delivery.

With assumed low delivery of *Pa-*IgY to the alveoli and no or very little growth of *P. aeruginosa* in blood cultures binding of *Pa-*IgY to *P. aeruginosa* is unlikely. However, there is a possibility of *Pa-*IgY binding isolated antigens that translocate to the blood stream. This would explain the difference seen in TNF-α. The intervention group had higher HR, CI and DO_2_ and lower SVRI. Interestingly, these circulatory changes are the opposite compared to when *Pa-*IgY is delivered intra-bronchially.^21^ Since increased HR can lead to both increased CI and increased DO_2_, it is likely that the difference in HR is causing the differences observed in CI and DO_2_. We also observed higher ΔpCO_2_/ΔcO_2_ in the intervention group, this might be explained by inadequacy of microcirculatory function. Lactate levels were lower in the intervention group, these findings might be explained by microcirculatory disturbances that are compensated by increased DO_2_ with lessened tissue hypoxia as a result. This is supported by the low values of ΔpCO2/ΔcO_2_, values below 1.4 mmHg are generally not linked to anaerobic metabolism.^20,29^ Lower levels of Hb and higher PaO_2_/FiO_2_-ratio in the intervention group could imply decreased endothelial damage in the intervention group with lower capillary leakage. Whether the circulatory-, inflammatory- and respiratory differences observed are beneficial for the intervention group are beyond the scope of this study. However, these findings are interesting and generate hypotheses for future studies.

This is, as far as we know, the first study to report the effect of *iv Pa*-Ig*Y,* a necessary step when studying a potential future treatment. Anti-canine parvovirus (CPV) IgY antibodies have been studied intravenously in dogs before, also without major adverse events.^30^ The use of a mechanically ventilated large animal model in an ICU-setting resembling clinical VAP is a strength of this study. This allows for complex physiological measurements and repeated blood- and tracheal sampling. The double blind and randomized design eliminates the risk of bias. While the number of animals is small it allow for major effects to be found. These experiments are cumbersome, expensive and limiting the number of animals is important for ethical reasons. Growth in pulmonary biopsies was heterogeneous, a different culture method such as culturing the entire right lower lobe might have produced more consistent concentrations of *P. aeruginosa*.

Research in this area should explore the effect of higher doses of *Pa*-IgY to treat pneumonia caused by *P. aeruginosa.* Detailed pharmacokinetic analyzes to examine the delivery of immunoglobulins to the alveoli in pneumonia should be performed as this would be applicable to all future studies on *iv* immunoglobulins in pneumonia. There have been studies where immunoglobulin G has been modified to increase delivery into the alveoli, the same method could possibly be applied to *Pa-*IgY In this study we have used anti-pseudomonas IgY, the effect of IgY on other pathogens causing pneumonia is unclear. Future studies should use IgY specific for other multi-resistant bacteria, such as *Staphylococcus aureus* or *Klebsiella Pneumoniae*, in their respective pneumonia models. This first study on *iv Pa-*IgY has not been designed to thoroughly investigate the safety profile of the drug, future research should examine this area further.

## Conclusions

In summary, in an anesthetized and mechanically ventilated porcine model of VAP, *Pa-*IgY at the dose used did not decrease concentrations of *P. aeruginosa* in the lower airways.
